# Building a biopsychosocial model of cancer-related fatigue: the BIOCARE FActory cohort study protocol

**DOI:** 10.1186/s12885-021-08831-3

**Published:** 2021-10-23

**Authors:** M. Chartogne, A. Leclercq, B. Beaune, S. Boyas, C. Forestier, T. Martin, V. Thomas-Ollivier, S. Landry, H. Bourgeois, O. Cojocarasu, V. Pialoux, O. Zanna, L. A. Messonnier, A. Rahmani, B. Morel

**Affiliations:** 1 Le Mans Université, Movement - Interactions - Performance, MIP, 4334, F-72000 Le Mans, EA France; 2 Nantes Université, Movement - Interactions - Performance, MIP, 4334 Nantes, EA France; 3 Elsan-Clinique Victor Hugo, Centre Jean Bernard, Le Mans, France; 4 Centre Hospitalier Le Mans (CHM), Le Mans, France; 5 Univ Lyon, University Claude Bernard Lyon 1, Inter-University Laboratory of Human Movement Biology, Team Atherosclerosis Thrombosis & Physical Activity, EA7424, Lyon, France; 6 Le Mans Université, VIPS2, EA4636, Le Mans, France; 7 Laboratoire Interuniversitaire de Biologie de la Motricité, Univ. Savoie Mont Blanc, 7424, F-73000 Chambéry, EA France

**Keywords:** Cancer-related fatigue, Breast cancer, Fatigability, Correlates of fatigue, Structural equation modeling, Follow-up

## Abstract

**Background:**

Cancer-related fatigue (CRF) is the most common side effect of cancer and cancer treatment. CRF prevalence is up to 50% in breast cancer patients and can continue several years after cancer remission. This persistent subjective sense of exhaustion is multifactorial. Numerous parameters have been evidenced to be related to CRF across biological, physical, psychological, social and/or behavioral dimensions. Although CRF has been studied for many years, the majority of previous studies focused on only one dimension, i.e., physical function. Moreover, few studies investigated CRF longitudinally with repeated measures. These are the two main obstacles that limit the understanding of CRF mechanisms. The purpose of this study is to create a biopsychosocial model of CRF with simultaneous and longitudinal anthropometric, clinical, biological, physical, psychological and sociological parameters.

**Methods:**

BIOCARE FActory is a multicentric prospective study that will consist of an 18-month follow-up of 200 women diagnosed with breast cancer. Four visits will be scheduled at diagnosis, after treatments, and 12 and 18 months after diagnosis. The same procedure will be followed for each visit. Each session will be composed of anthropometric data collection, a semi-structured interview, cognitive tests, postural control tests, neuromuscular fatigability tests and a cardiorespiratory fitness test. Clinical and biological data will be collected during medical follow-ups. Participants will also complete questionnaires to assess psychological aspects and quality of life and wear an actigraphy device. Using a structural equation modeling analysis (SEM), collected data will build a biopsychosocial model of CRF, including the physiological, biological, psychological, behavioral and social dimensions of CRF.

**Discussion:**

This study aims to highlight the dynamics of CRF and its correlates from diagnosis to post treatment. SEM analysis could examine some relations between potential mechanisms and CRF. Thus, the biopsychosocial model will contribute to a better understanding of CRF and its underlying mechanisms from diagnosis to the aftermaths of cancer and its treatments.

**Trial registration:**

This study is registered at ClinicalTrials.gov (NCT04391543), May 2020.

## Background

Cancer and cancer treatments induce various side-effects, the most reported being cancer-related fatigue (CRF). CRF is defined as ‘a distressing persistent subjective sense of physical, emotional, and/or cognitive tiredness or exhaustion related to cancer or cancer treatment that is not proportional to recent activity that interferes with usual functioning’ [1]. In breast cancer patients, CRF prevalence is up to 50% and can persist two years after cancer remission for 30% of patients and 5 years after for 20% [2]. CRF is extremely disturbing to quality of life and can decrease survival (overall and recurrence-free survival) [3]. Numerous parameters have been evidenced to be related to CRF among biological, physical, behavioral, psychological and/or social dimensions [4] (Table 1). Below is a summary of the main correlates of CRF (for extensive reviews see 5,6,7).

**Table 1:** Hypothesized mechanisms of CRF and corresponding dimensions (adapted from McNeely and Courneya 2010)

Physiological	Biological	Psychological	Behavioral	Social
• Muscular strength • Muscular endurance • Cardiopulmonary fitness • Body composition	• Inflammatory response • Metabolic function • Endocrine function • Immune function	• Anxiety • Depression • Distress • Cognition	• Sleep quality and quantity • Appetite	• Social interactions • Positive reinforcement

Anthropometric data such as BMI or age have been, respectively, positively and negatively correlated to CRF severity [5–8]. Clinical data such as treatment type and disease stage have been associated with higher severity of CRF [9–11]. This relationship, however, remains unclear, and Bower et al., [12] concludes that treatment-related factors accounted for only a small portion of CRF.

Biological parameters extracted from blood samples, such as inflammation and anemia, have been related to CRF in breast cancer survivors through elevated levels of pro-inflammatory cytokines (e.g., TNF-α, IL-6) and reduced hemoglobin levels, respectively [13, 14].

Regarding physical parameters, CRF patients are more susceptible to sarcopenia, a substantial loss of skeletal muscle, thereby altering muscle strength and endurance [15]. This decrease in the force-generating capacity of the neuromuscular system during exercise, or neuromuscular fatigability, is higher in cancer patients suffering from CRF and is probably controlled by a specific central etiology [16–20]. Cardiorespiratory deconditioning is also linked to CRF; VO_2peak_ and power output at the lactate threshold have been correlated to CRF’s severity in cancer survivors [21], possibly due to the cardiac toxicity of some chemotherapies [22].

Behavioral parameters such as sleep disturbances are highly prevalent (up to 70%) in patients experiencing CRF [23]. Recent objective measures of sleep showed that onset latency, wake time at night, and sleep efficiency were correlated with CRF severity [24]. Lastly, duration and intensity of spontaneous daily physical activities are also related to CRF, as patients show an increase in leisure time and low intensity activities (< 2.5 metabolic equivalent task, METs) compared to those without CRF [25].

The relationship to psychological dimensions is also well established. Using specific questionnaires, anxiety and depression were investigated in breast cancer patients across many years and were strongly correlated with CRF severity [26]. Coping strategies, particularly catastrophizing (a lack of confidence and an expectation of negative outcomes), may be associated with CRF [27]. Furthermore, women with breast cancer may undergo surgical mastectomy in addition to chemotherapy, which impacts body image, self-esteem and may lead to depressive symptoms [28, 29]. Cognitive processes, such as attention, concentration and memory, are affected before and during treatments and related to CRF [30]. These cancer-related cognitive impairments have been frequently attributed to chemotherapy neurotoxicity and dubbed “chemo fog” or “chemobrain” [31]. As part of the social dimension of CRF, social networks and support were studied using questionnaires and interviews. Generally, the lack of social support was identified as a factor of fatigue in chronic fatigue syndrome [32]. Among breast cancer patients, those who reported lower levels of social support had elevated CRF [33].

Despite the numerous studies investigating CRF correlates, including their potential mechanisms and the well-known multidimensional nature of CRF, the majority of previous studies have remained mainly focused on a specific dimension (e.g., biological or psychological). This is a significant limitation to understanding the relationships between all of the known correlates of CRF. There are, however, a few studies that have combined some of the above-mentioned dimensions in order to investigate CRF mechanisms. For example, Stone et al., [34] designed a model using a multiple linear regression, explaining 56% of CRF variance including anxiety-depression, dyspnea and pain, and a disease burden score. More recently, Lockefeer et al., [26] used depressive symptoms, sleep quality and CRF before diagnosis (or primary surgical treatment) in breast cancer patients to predict CRF at 24 months. Only CRF before diagnosis was a significant predictor of CRF two years later and explained 33% of CRF variance. Humpel et al., [35] also investigated the relationship between sleep disturbances, CRF and physical activity in patients with breast cancer. Their CRF prediction model included sleep quality and total physical activity and resulted in a 46% CRF variance prediction. Nevertheless, they focused exclusively on the behavioral dimensions of CRF. Recently, CRF prediction has been studied by combining neuromuscular, emotional and behavioral dimensions [20]. The results evidenced that a model including anxiety-depression, sleep disturbances and neuromuscular fatigability explained 56% of CRF variance. CRF variances described by the above-mentioned studies ranged from 33 to 56%, leaving unexplained variations which could be related to dimensions not yet considered.

Most studies investigating CRF were cross-sectional and thus cannot account for the dynamics of these mechanisms, and few studies implemented longitudinal follow-ups. Some longitudinal studies have focused on the psychological dimension and their results are unanimous in asserting that anxiety before treatment was a strong predictor of subsequent CRF in breast cancer patients [26, 36, 37]. At best, these various studies combine only two different dimensions (behavioral and psychological, biological and psychological, clinical and psychological, respectively). Only Bower et al., [5] have led a longitudinal study in breast cancer patients that combines more than two dimensions of CRF, from diagnosis to 18 months post treatment. They have investigated biological, demographic, social, clinical and psychological dimensions. Unfortunately, only baseline results are currently available. They reveal that younger age, lower educational level, lower disease stage and history of childhood maltreatment were found to be significant predictors of CRF.

Recent studies have highlighted the necessity to develop longitudinal and multidimensional researches in order to identify potential mechanisms explaining CRF [8, 38]. Therefore, the purpose of this longitudinal study is to build a biopsychosocial model of CRF by simultaneously investigating anthropometric, clinical, biological, physical, psychological and sociological parameters using structural equation modeling (SEM).

## Methods/design

### Study population

Two hundred women newly diagnosed with breast cancer by an oncologist and satisfying inclusion and exclusion criteria (Table 2) will be informed of the study protocol. If interested, patients will receive an information letter from their oncologist and will be given the opportunity to ask any questions pertaining to it. After a 48 h period of reflection, study coordinators will phone the patients to confirm their participation, obtain a written consent form and schedule their first visit.

**Table 2:** Inclusion and exclusion criteria

Inclusion criteria	• Patients care by chemotherapy or radiotherapy in Clinique Victor Hugo-Centre Jean Bernard, Le Mans, France or in Centre Hospitalier du Mans, Le Mans, France
	• Patients with breast cancer diagnosis (Stage I to IIIc)
	• Aged ≥18 and ≤ 80 years
	• Approval received from oncologist
	• ECOG Performance Status ≤2
	• French speaking (able to understand questionnaires and instructions related to study procedures)
	• Written informed consent obtained
**Exclusion criteria**	• Comorbidities related to fatigue symptoms
	• Polyneuropathy, amyotrophy or myasthenic syndrome diagnosis
	• Contraindications to physical activity or to experimental procedures
	• Antidepressants, psychostimulants, psychotropics, antiépileptics or benzodiazepine based treatment
	• Previous or current psychosis, bipolarity or severe depression symptoms
	• History of chronic fatigue, stroke or musculoskeletal disorders
	• Participant is pregnant

### Study design

**Fig. 1:**
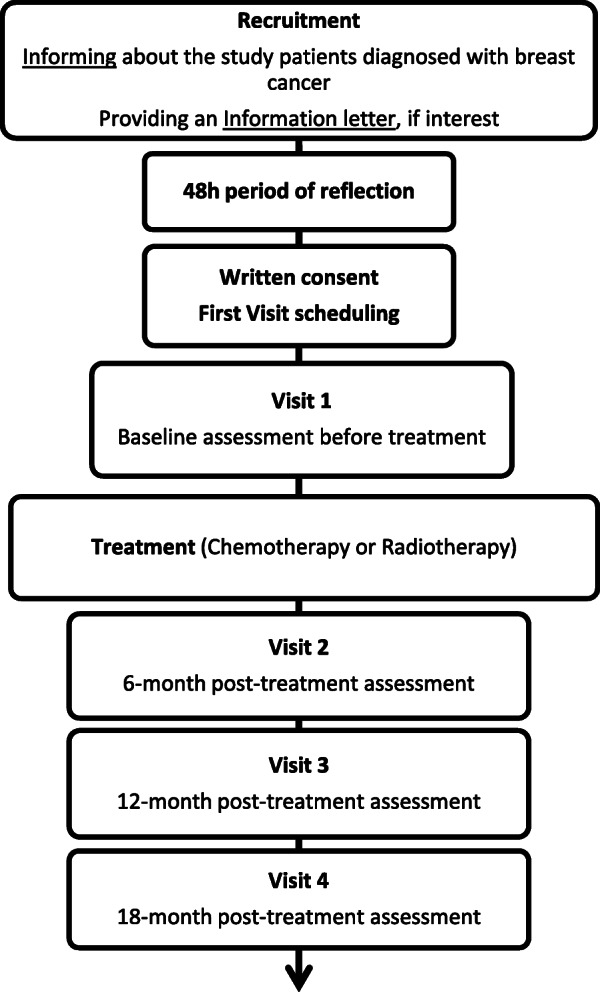
Flowchart of the study design

This prospective multicentric (Clinique Victor Hugo-Centre Jean Bernard, Le Mans, France and Centre Hospitalier du Mans, Le Mans, France) study will consist of an 18 month follow-up of a women cohort with breast cancer. Four visits will be scheduled (the study timeline is presented in Fig. 1). Detailed patient assessments will be performed before treatment begins (visit 1: diagnosis); during the week after completion of the first-line treatment, or 6 months after diagnosis, whichever comes first (visit 2); and then 12 and 18 months after diagnosis (visit 3 and 4, respectively). This study has been approved by the French ethics committee of human research CPP SUD EST VI (IDRCB: 2019-A02525–52) and will be performed according to the Declaration of Helsinki. Furthermore, this study protocol has been written in accordance with the SPIRIT guidelines (SPIRIT Checklist provided in Additional file 1) and is registered in a database (ClinicalTrials.gov, NCT04391543, May 2020). In order to longitudinally assess the evolution of biopsychosocial dimensions of CRF, the same procedure will be followed for each visit. Each session will last 1.5 h and will be composed of anthropometric data collection, a semi-structured interview, cognitive tests, postural control tests, neuromuscular fatigability tests and a cardiorespiratory fitness test. Clinical and biological data will be collected during medical follow-ups. In addition, participants will be asked to wear an actigraphy device (received by mail one week prior to each experimental session) and to complete questionnaires to assess psychological aspects and quality of life.

### Anthropometric data

Body height, body mass, lower limb fat mass (using impedancemetry) and calf circumference (CC) will be measured. CC will be recorded to assess the dominant lower limb volume using the truncated cones technique [39] by dividing the lower limb volume into a series of segments. Then, the lower limb volume will be used to normalize maximal strength of participants. CC under 31 cm will serve as a clinical indicator of sarcopenia [40].

### Semi-structured interview

To investigate the social dimension of CRF, a semi-structured interview will be conducted (duration ~ 30 min). Demographic (e.g., age, gender, socio-professional category, residence area, income) and sociability (e.g., family, friendly, professional) will be addressed. The interview will be introduced by an open question on a typical week to then develop the sociability. The entire interview will be audio recorded, fully transcribed and then will be the subject of a lexicographical treatment for rating quantity and intensity of sociability (on a scale from − 5 to 5). In order to build a typology of patients’ sociability, researchers will also assess the level of social ties’ perception. Lastly, family, friendships, professional sociabilities and income will be considered in the SEM analysis.

### Cognitive tests

The Montreal Cognitive Assessment (MoCA), the Trail Making Test (TMT) and the Stroop test will be completed by participants to assess cognitive functions. The MoCA is separated into several tasks including visuospatial/executive functioning, naming, memory, attention, language, abstraction, delayed recall and orientation. For example, the participants will be asked to connect numbers and letters in a defined sequence, count backwards from 100 by increments of 7, draw different figures and perform word associations. The MoCA total score (/30) is obtained by summing the scores from each item; a cut-off score below 26 is an indicator of mild cognitive impairment [41]. The TMT, evaluating processing speed and cognitive flexibility, consists of connecting numbers in ascending order (Part 1) and then in connecting numbers and letters in both ascending and alphabetical order (Part 2), as fast as possible [42]. Time needed to complete each part and number of mistakes will be recorded to assess performance. The Stroop test is based on color recognition (i.e., red, green, yellow and blue) with interference in three parts, each composed of 6 lines with 4 items per line (colored sticker or word). The first part is composed of colored stickers (control), the second has colored words (low interference) and the last part has color-words printed in a color not denoted by the name (high interference). For each part, participants will be advised to name the ink color for each word, regardless of the semantic content, as quickly and accurately as possible. The time to complete each part is measured to attribute an interference score used to assess response inhibition, which has been related to cognitive functioning in daily life [43]. The TMT and the Stroop test will be implemented on Inquisit software (Millisecond Software, LCC, Seattle, USA). For SEM analysis, MoCA total scores (/30), TMT time (s) and Stroop interference scores (%) will be considered.

### Postural tests

Participants will be asked to stand up barefoot, as still as possible, during 60 s on a pressure distribution measurement platform (FDM-S, zebris Medical GmbH, Isny, Germany). Feet position will be standardized using markings to keep both heels spaced apart by 10 cm and an angle of 15° between both feet. Participants will be advised to keep arms alongside the body during recording. Four conditions composed of two trials of a simple task and two trials of a dual task (counting backwards by 2 from a number close to 100) with eyes open or closed will be counterbalanced and interspersed with a 2 min rest period. In eyes open conditions, participants will be asked to stare at a visual marker placed 2 m in front at eye level. For the dual task, participants will be asked to count as fast as possible whilst remaining as immobile as possible. The numbers of answers and errors will be recorded. Temporal analysis will be performed in order to investigate anterior-posterior and medial-lateral center of pressure (COP) distance, mean and maximal COP velocities and 95% confidence ellipse area. In addition, the frequency domain measures (relative power in < 0.5 Hz; 0.5–1.5 Hz; > 1.5 Hz frequency bands) will be computed. 95% confidence ellipse area (mm^2^) will be considered in the SEM to represent the physical function latent variable, while maximal COP velocities (mm/s) will be considered representative of the emotional function latent variable, as it is a relevant hallmark of depression-related psychomotor retardation [44].

### Neuromuscular fatigability test

**Fig. 2:**
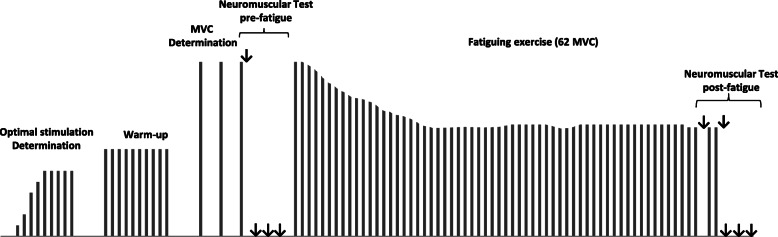
Schematic illustration of the fatiguing exercise and neuromuscular assessments. ↓ represents electrical stimulation

Throughout this test, participants will remain in prone position on a patient table, with a fully-extended knee and an ankle angle of 90°, their foot securely blocked at the metatarsal level in a custom–made device enabling isometric strength measurement with a load cell (LSB350, Futek, Irvine, USA). Firstly, optimal electrical stimulation intensity will be determined to set the supramaximal intensity used during the subsequent neuromuscular assessments by progressively increasing the current (from 20-mA to 200-mA, with a 20-mA increment) until there is no further increase in the evoked isometric twitch response. The last intensity obtained will be further increased by 20% to ensure stimulus supramaximality. All electrical stimulations will be delivered with a constant current (Digitimer DS7A-H, Hertfordshire, UK) using square-wave stimuli of 200 μs duration with a maximal voltage of 400-V and via rectangular self-adhesive electrodes (5 × 10 cm, Compex). The cathode will be placed over the gastrocnemii (~ 5 cm distal to the popliteal fossa) and the anode over the soleus (~ 10 cm proximal to the calcaneus). Secondly, participants will accomplish a standardized warm-up (10 isometric contractions of 4 s at 50% of their maximal strength with 4 s of recovery in between), followed by 2-min of rest and then by the maximal voluntary contraction (MVC) measurement (two MVC of 4 s separated by 2 min; if the difference between these MVCs is superior to 5%, a third one will be performed). The higher peak force produced will be considered as the MVC in non-fatiguing conditions. Next, pre-fatigue neuromuscular functions will be assessed on a third MVC, using a 100-Hz doublet during the force plateau and a stimulation sequence on the relaxed muscles beginning 2 s after the end of contraction: a 100-Hz doublet, a 10-Hz doublet and a simple stimulation, interspersed by 3 s. The fatiguing exercise will be composed of 62 isometric MVC in ankle plantar flexors with the dominant leg (Fig. 2). Each MVC will last 4 s with 1 s rest; duty cycle will be ensured using a metronome with visual and sound signals. To avoid pacing strategies, participants will not be informed of the time remaining or the number of MVC performed [45]. Investigators will use verbal encouragements for participants to contract as strong as possible during MVC. The post-fatigue neuromuscular functions will be assessed on the last MVC of the fatiguing exercise (62nd) with the same stimulation procedure as in pre-fatigue condition. On the 60th MVC, neuromuscular functions will also be tested but only with two 100-Hz doublets (during the force plateau and on the relaxed muscles 2 s after the MVC end).

Peak force occurring during each 4-s MVC set will be recorded and the force-time relationship asymptote (F_A_; expressed in percentage of the MVC in non-fatiguing conditions) will be used to represent a neuromuscular fatigability threshold, above which fatigability drastically sets in [20, 46, 47]. Then, voluntary activation (VA) and evoked forces by a 100-Hz doublet (Db100) will be determined using the interpolated twitch technique [48] and expressed in pre-post fatiguing test differences, normalized to pre-fatiguing test values. F_A_ (% MVC), VA (%) and Db100 (%) will be considered in the SEM.

### Cardiorespiratory fitness test

After a five-minute recovery period, a blood drop (10 μL) will be taken from the earlobe and analyzed extemporaneously (within 15 s) to obtain a resting blood lactate concentration ([Lactate]_b_) (Lactate Scout 4, EKF diagnostics, Cardiff, UK). Then, participants will perform a submaximal incremental exercise on a cycle ergometer (model 928E, Monark, Varberg, Sweden), with saddle and handlebar heights adjusted for each patient. Throughout the test, participants will be encouraged to maintain a constant cadence (approximately 60 rpm) and heart rate (HR) will be recorded with a heart rate monitor (HRM-Dual, Garmin, Olathe, USA). After a 3-min standardized warm-up at a rate of perceived exertion (RPE) of 8–9 (Borg 6–20 scale), the test will start at 20 W. Every 2 min [Lactate]_b_ will be measured as previously described and then intensity will be increased by 10 W steps. Just before the end of the step, participants will indicate RPE (Borg 6–20 scale). Exercise will be stopped as soon as i) [Lactate]_b_ ≥ 4 mmol. L^− 1^; ii) participant RPE > 15; or iii) participant will no longer be able to maintain the 60 rpm cadence. Participants will be able to interrupt the exercise when they wish to do so, particularly in the case of nausea, chest pain or dyspnea. This test will be used to determine the physiological and biomechanical parameters (i.e., HR, power output associated with the first lactate threshold (LT1; defined as the first inflection point in the lactate concentration ([Lactate]_b_) curve). Power output at LT1 (W) will be considered in the SEM.

### Clinical and biological data

As experimental sessions will accompany medical follow-ups, clinical and biological data will be also recorded. Cancer stage and details related to treatments (e.g., type, duration and dose) will be recorded in a medical file by their oncologist. Venous blood samples will be collected by medical staff. Plasma will be obtained after a 10 min centrifugation at 4 °C, then divided into aliquots and stored at − 80 °C until analysis. Following assays will be performed on plasma for the assessment of inflammation (IL-6, TNFɑ, IL-8, IL-1β). Abdominal computed tomography (CT) images at the L3 level will be taken by medical staff on two occasions (Visit 1 and Visit 2). The surface of the muscular tissues will be selected according to the CT Hounsfield unit and normalized to stature in order to calculate the lumbar skeletal muscle index (LSMI). A cut-off value (LSMI < 38.5cm^2^/m^2^) will be used to characterize sarcopenia [49]. Cancer stage and treatment; sarcopenia; and IL-6, IL-1β, TNFɑ (pg/mL) concentrations will be considered in the SEM.

### Participant-reported outcomes

One week prior to each experimental session, participants will receive by post self-assessment questionnaires about quality of life (EORTC QLQ-C30), CRF (EORTC QLQ-FA12), coping strategies (Brief Cope) and anxiety-depression symptoms (The Hospital Anxiety and Depression Scale - HADS). Participants will be asked to complete questionnaires, alone in quiet conditions, and bring it back the day of the experimental session. The subsequent instructions will be provided: “Please answer all questions yourself by circling the number that best applies to you. The information provided will remain strictly confidential. Take as much time as necessary. There are no “right” or “wrong” answers.”

The EORTC QLQ-C30 Fatigue scale score (FA item) will be used to assess the general degree of CRF (ranging from 0 to 100; with higher levels indicating a greater degree of CRF), with a threshold for clinical importance of 39 [50]. EORTC QLQ-FA12 subscales will be used to assess physical, cognitive and emotional dimensions of CRF [51]. The HADS subscale scores (/21) will be used to assess anxiety and depression, respectively [52]. In the SEM, general degree of CRF (%); dimensions of CRF (%) (physical, cognitive and emotional); coping scores (/8); and anxiety and depression scales (/21) will be considered.

### Actigraphy

One week prior to each experimental session, participants will be asked to wear a portable device (eTact®, BodyCap, Caen, France) containing a tri-axial accelerometers enabling to record every acceleration from the body (sampling frequency: 25 Hz, measurement range: 0.1-2G, sample measurement: 1 min). They will be advised to wear the device continuously for 7 days (days and nights) regardless of daily activities. Sleep characteristics will be obtained using the eTact® Analysis software (BodyCap, Caen, France), which assess the individual sleep quality based on the actigraphy data collected over selected periods. Considered sleep parameter will be sleep time (i.e, duration of effective sleep period) and sleep efficiency (i.e., percentage of time spent sleeping during the rest period). From the software, total activity duration during wake period will be calculated as the accumulated time in each activity intensity band (i.e., low, moderate and intense). In addition, participants will be asked to complete a sleep diary (including estimated sleep onset and offset hour) during the actigraphy measurements. Total activity duration (min) and sleep efficiency (%) will be considered in the SEM.

### Sample size

A sample size of *n* = 200 patients has been chosen on the basis of resource constraints [53]. 200 correspond to a 15% inclusion rate of the patients meeting the inclusion criterion during a 24 months inclusion period and including a 20% potential drop-out rate. Considering this sample size, a sensitivity power analysis performed with G-power (*F* tests family, linear multiple regression, *R*^*2*^ increase) to estimate the smallest effect size we could detect with 90% power regarding our main independent variable (i.e., CRF) at the level of analysis with the lowest power (i.e., level 2, between-person level). This analysis, with a power of 0.90, an α level of 0.05 and 6 predictors (i.e., 6 latent variables), revealed that the smallest effect size we could detect with 90% power, at this level of analysis, is *f*^*2*^ = 0.09, which correspond to a small to medium effect.

### Statistical analysis

Hypotheses will be addressed by constructing a biopsychosocial model of the relationships among biological, physical, emotional, cognitive and social dimensions of CRF using multilevel SEM. SEM is particularly well-suited to this type of analysis because it simultaneously accounts for multiple interactive relationships among variables, easily handles multiple sources of variance, and permits testing of hypothesized directional relationships [54, 55]. Consequently, the results of SEM analyses facilitate inferences regarding the relationships among variables [56]. Relations between the manifest variables and their underlying latent constructs in a hypothesized biopsychosocial model predicting CRF are presented in Table 3. The model will be tested with the maximum likelihood method using Lavaan *R* package for SEM [57]. A primary cross-sectional analysis will be completed using data collected at each visit, and then subsequently by using the longitudinal data set. The models’ fit was assessed by examining the minimum discrepancy (CMIN/DF), the probability level (*p*-value), the Bentler-Bonett normed fit index (NFI), the comparative fit index (CFI), the Tucker–Lewis-Index (TLI), and the root-mean-square error of approx- imation (RMSEA). A satisfactory model fit is indicated by a CMIN/DF ratio below 2.00 [58], a *p*-value over 0.05 [59], a NFI over 0.95 [60], a TLI over 0.90 [61], a CFI over 0.93 [62], and a RMSEA below 0.05 [63]. All non-significant paths were deleted according to the methods described by MacCallum [64].

**Table 3:** Latent and manifest variables included in the model built by SEM

Latent variable	Manifest variable	Test
**Social function**	Family sociability	Semi-structured interview
	Friendly sociability	Semi-structured interview
	Professional sociability	Semi-structured interview
	Income	Semi-structured interview
**Cognitive function**	MoCA total score	MoCA
	TMT time	TMT
	Interference score	Stroop
**Physical function**	95% confidence ellipse area	Postural test
	F_A_	Neuromuscular fatigability test
	VA	Neuromuscular fatigability test
	Db100	Neuromuscular fatigability test
	Power output at LT1	Cardiorespiratory fitness test
	Sleep efficiency	Actigraphy
	Total activity duration	Actigraphy
	Sarcopenia	Abdominal CT at the L3 level
**Biological function**	Cancer stage	Medical file
	Cancer treatment	Medical file
	IL-6 concentration	Blood sample
	IL-1β concentration	Blood sample
	TNFɑ concentration	Blood sample
**CRF**	FA item score	EORTC QLQ-C30
	Physical subscale	EORTC QLQ-FA12
	Cognitive subscale	EORTC QLQ-FA12
	Emotional subscale	EORTC QLQ-FA12
**Emotional function**	Coping score	Brief Cope
	Anxiety scale	HADS
	Depression scale	HADS
	Maximal COP velocity	Postural test

## Discussion

American and European guidelines [65–67] recommend screening for CRF using self-reported questionnaires [68]. Even though this method remains simple and easily feasible for both clinician and patients, this assessment method does not enable the understanding and management of underlying mechanisms. Despite that CRF has been studied for decades, and recent studies have recognized that CRF is multifactorial and may be influenced by a variety of mechanisms [8, 38, 69], few longitudinal and multidimensional studies have been implemented. This is one of the main obstacles to the assessment and management of CRF. Therefore, our longitudinal study is designed to build a model by examining longitudinally biopsychosocial correlates of CRF.

The greatest challenge in designing this study was to select the most pertinent and suitable parameters/variables for each of the biopsychosocial dimensions in order to remain feasible for fragile patients. As it is not possible to measure all parameters potentially involved in CRF, we had to exclude some dimensions such as pain or nutrition that would have otherwise been pertinent [34, 70]. All parameters included are among those most related to CRF, according to the literature.

All patients with localized breast cancer will be included, inherently incorporating various conditions of cancer stages and treatment. Although this may result in a high level of variability and statistical noise, we deemed it necessary to build a real life model that can be later used in clinical practice. Furthermore, with a prevalence up to 50% for CRF [2], it is reasonable to believe that a proportion of participants will not be suffering from clinically important CRF. This could be perceived as a limitation when studying CRF. However, because clinical relevance is based on cut-off values, some patients could potentially be misclassified and we think that CRF should be understood as a continuum. Moreover, even mild CRF can have an impact on patient quality of life.

The BIOCARE FActory study is expected to highlight the dynamics of CRF and its correlates from diagnosis through post-treatment. SEM analysis will hypothesize relations between latent variables (e.g., physical function), assessed through observed variables (e.g., force-time asymptote, voluntary activation) and the CRF variable. Results of the present biopsychosocial model will greatly contribute to a better understanding of CRF and its underlying mechanisms, from diagnosis to the aftermaths of cancer and treatments. They could also be applied to improving interventions for CRF management, notably in supportive care, thanks to a better understanding of CRF mechanisms.

## Data Availability

Not applicable (the current manuscript contains no data).

## Abbreviations

**CC**: Calf Circumference
**COP**: Centre Of Pressure
**CPP**: Committee for the Protection of Persons
**CRF**: Cancer-Related Fatigue
**CT**: Computed Tomography
**ECOG**: Eastern Cooperative Oncology Group
**EORTC**: European Organisation for Research and Treatment of Cancer
**F**_**A**_: Force-time relationship Asymptote
**HADS**: The Hospital Anxiety and Depression Scale
**HR**: Heart Rate
**IL**: Interleukin
**LSMI**: Lumbar Skeletal Muscle Index
**LT1**: First Lactate Threshold
**MET**: Metabolic Equivalent Task
**MoCA**: Montreal Cognitive Assessment
**MVC**: Maximal Voluntary Contraction
**RPE**: Rate of Perceived Exertion
**SEM**: Structural Equation Modelling
**TMT**: Trail Making Test
**TNF**: Tumor Necrosis Factor
**VA**: Voluntary Activation
